# Increasing TRPV4 expression restores flow-induced dilation impaired in mesenteric arteries with aging

**DOI:** 10.1038/srep22780

**Published:** 2016-03-07

**Authors:** Juan Du, Xia Wang, Jie Li, Jizheng Guo, Limei Liu, Dejun Yan, Yunyun Yang, Zhongwen Li, Jinhang Zhu, Bing Shen

**Affiliations:** 1 Department of Physiology, School of Basic Medicine, Anhui Medical University, Hefei, Anhui 230032, China; 2 Central laboratory of molecular and cellular biology of School of Basic Medicine, Anhui Medical University, Hefei, Anhui 230032, China; 3 Department of Physiology and Pathophysiology, Peking University Health Science Center, Peking 100191, China

## Abstract

The flow-stimulated intracellular Ca^2+^ concentration ([Ca^2+^]_i_) rise in endothelial cells is an important early event leading to flow-induced blood vessel dilation. Transient receptor potential vanilloid subtype 4 (TRPV4), a Ca^2+^-permeable cation channel, facilitates the flow-stimulated [Ca^2+^]_i_ rise. To determine whether TRPV4 is involved in age-related flow-induced blood vessel dilation impairment, we measured blood vessel diameter and nitric oxide (NO) levels and performed Ca^2+^ imaging, immunoblotting, and immunostaining assays in rats. We found that the flow-induced and TRPV4 activator 4α-PDD-induced dilation of mesenteric arteries from aged rats were significantly decreased compared with those from young rats. The flow- or 4α-PDD-induced [Ca^2+^]_i_ rise was also markedly reduced in primary cultured mesenteric artery endothelial cells (MAECs) from aged rats. Immunoblotting and immunostaining results showed an age-related decrease of TRPV4 expression levels in MAECs. Additionally, the 4α-PDD-induced NO production was significantly reduced in aged MAECs. Compared with lentiviral GFP-treated aged rats, lentiviral vector delivery of *TRPV4* increased TRPV4 expression level in aged MAECs and restored the flow- and 4α-PDD-induced vessel dilation in aged mesenteric arteries. We concluded that impaired TRPV4-mediated Ca^2+^ signaling causes endothelial dysfunction and that TRPV4 is a potential target for clinical treatment of age-related vascular system diseases.

Shear stress generated from blood flow is an important physiological stimulus that triggers endothelial cell (EC)-mediated vascular dilation^1^. In this process, Ca^2+^ signaling is also a well-documented component^2^,^3^,^4^. An increase in intracellular Ca^2+^ concentration ([Ca^2+^]_i_) evokes the synthesis and release of endothelial vasodilators, including nitric oxide (NO), prostacyclin, and endothelium-derived hyperpolarizing factors (EDHFs), which subsequently act on smooth muscle, leading to vessel dilation. Several studies have demonstrated that the transient receptor potential vanilloid subtype 4 (TRPV4), a Ca^2+^ permeable cation channel, facilitates the shear stress-mediated [Ca^2+^]_i_ increase^2^,^5^,^6^,^7^,^8^,^9^. Shear stress activates TRPV4 channels in TRPV4-overexpressing HEK293 cells, human umbilical vein endothelial cells, or renal epithelial cells^5^,^6^,^10^. Moreover, TRPV4 forms a channel complex with TRPC1, TRPP2, or both to mediate Ca^2+^ influx^9^. Elimination of TRPV4 in TRPV4 knockout (−/−) mice significantly reduces endothelium-dependent vascular dilation initiated by blood flow and slightly lowers baseline mean arterial pressure^11^.

Impaired endothelial function is considered a primary factor in aging, and it is a very early and important event leading to cardiovascular disease, such as vascular bed disorder, reported in both elderly animals and humans^12^,^13^,^14^,^15^,^16^. Without the presence of other cardiovascular risk factors, aging serves as an independent factor associated with endothelial dysfunction^17^. The current understanding of the mechanism underlying age-dependent endothelial dysfunction focuses on reduced NO bioavailability, enhanced oxidative stress, increased vasoconstrictor factor activity, and the development of a low-grade pro-inflammatory environment^18^. However, age-dependent changes of Ca^2+^ channels in ECs and the corresponding flow-stimulated [Ca^2+^]_i_ increase are largely uncharacterized. In the present study, we investigated the role of TRPV4 in flow-induced dilation impairment with aging and the potential value of TRPV4 in the clinical treatment of aging-related vascular system diseases.

## Results

### Changes in flow-induced vessel dilation and mesenteric artery ECs [Ca^2+^]_i_ rise with aging

**Figure 1:**
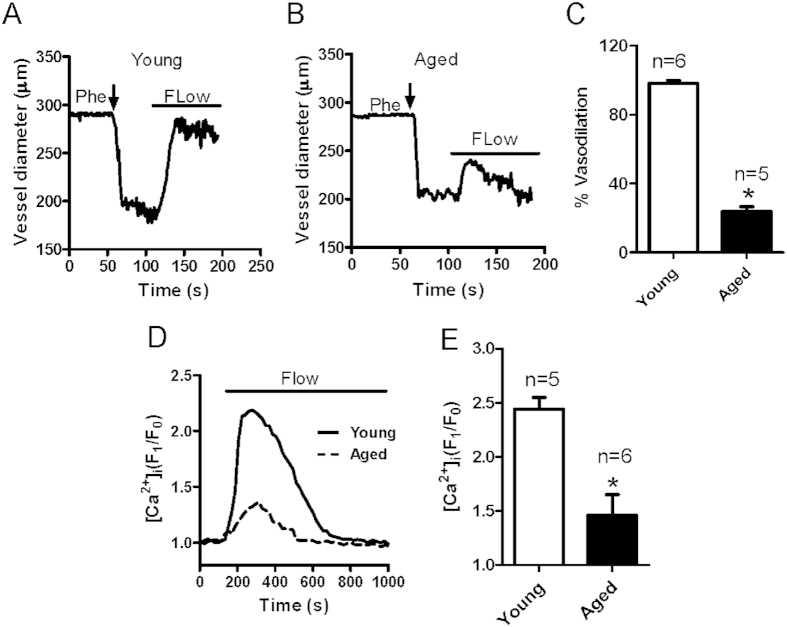
Age-related changes in flow-induced vessel dilation and intracellular Ca^2+^ concentration ([Ca^2+^]_i_) rise in mesenteric artery endothelial cells (MAECs). (**A**–**C**) Representative traces showing mesenteric artery dilation in response to flow challenge in young (**A**) and aged (**B**) rats. Vessels were preconstricted with phenylephrine (Phe) to approximately 60% of the preconstricted diameter. Solid bars above traces show the period of the flow. (**C**) Summarized data of peak flow dilation in young and aged vessels. (**D**) Representative traces of the flow-induced [Ca^2+^]_i_ rise in primary cultured MAECs derived from young and aged rats. (**E**) Summarized data showing the flow-induced [Ca^2+^]_i_ rise in young and aged MAECs. Values are mean ± SE (n = 5–6); ^*^P < 0.05, young (3 months old) vs. aged (22 months old) rats.

Flow-induced dilation was measured in rat mesenteric arteries derived from young (3 months old) and aged (22 months old) rats. Endothelium-intact mesenteric arteries were preconstricted to approximately 60% of their initial diameter using 1–3 μM phenylephrine (Phe). After stabilization, intraluminal flow was induced by a 10 mmHg pressure difference to elicit vascular dilation. We found that dilation was greatly reduced in mesenteric arteries derived from aged rats (Fig. 1A–C). Because flow-induced dilatation correlates with endothelium function^4^, the decreased flow response in aged blood vessels reflects an impairment in EC function with advancing age. Ca^2+^ signaling is crucial to the function of ECs for regulating vessel dilation. To determine whether the flow-induced [Ca^2+^]_i_ rise changes in aged mesenteric arteries ECs (MAECs), primary cultured MAECs were seeded on rectangular coverslip and mounted in a parallel flow chamber, where flow evoked a transient increase in [Ca^2+^]_i_. We found that the flow-induced [Ca^2+^]_i_ rise in MAECs derived from aged rats was significantly lower than that in MAECs from young rats (Fig. 1D,E). Therefore, this impaired flow-induced Ca^2+^ signaling in ECs may decrease flow-induced vessel dilation.

### Functional roles of TRPV4 in the flow-induced vessel dilation and MAEC [Ca^2+^]_i_ rise with aging

**Figure 2:**
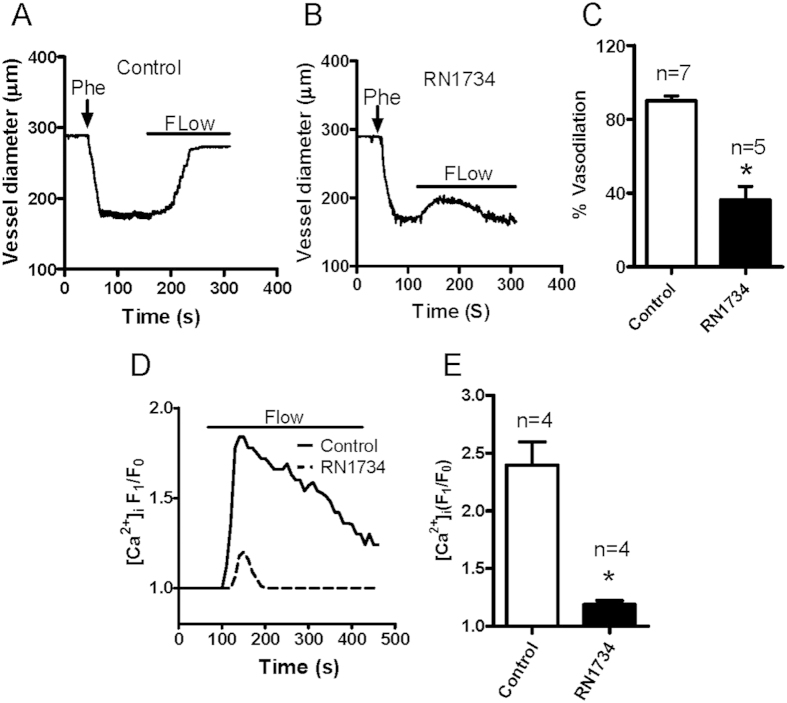
TRPV4 antagonist inhibits flow-induced mesenteric artery dilation and intracellular Ca^2+^ concentration ([Ca^2+^]_i_) rise in mesenteric artery endothelial cells (MAECs). (**A,B**) Representative traces showing flow-evoked vessel dilation in control (**A**) or TRPV4 inhibitor RN1734-pretreated (**B**) mesenteric arteries. Vessels were preconstricted with phenylephrine (Phe) to approximately 60% of the preconstriction diameter. Solid bars above traces show the period of the flow. (**C**) Summarized data of the peak flow dilation in control and RN1734-pretreated vessels. (**D**) Representative traces of the flow-induced [Ca^2+^]_i_ rise in control or RN1734-pretreated MAECs. (**E**) Summarized data showing the flow-induced [Ca^2+^]_i_ rise in control and RN1734-pretreated MAECs . Values are mean ± SE (n = 4–7); ^*^P < 0.05, control vs. RN1734 pretreatment.

**Figure 3:**
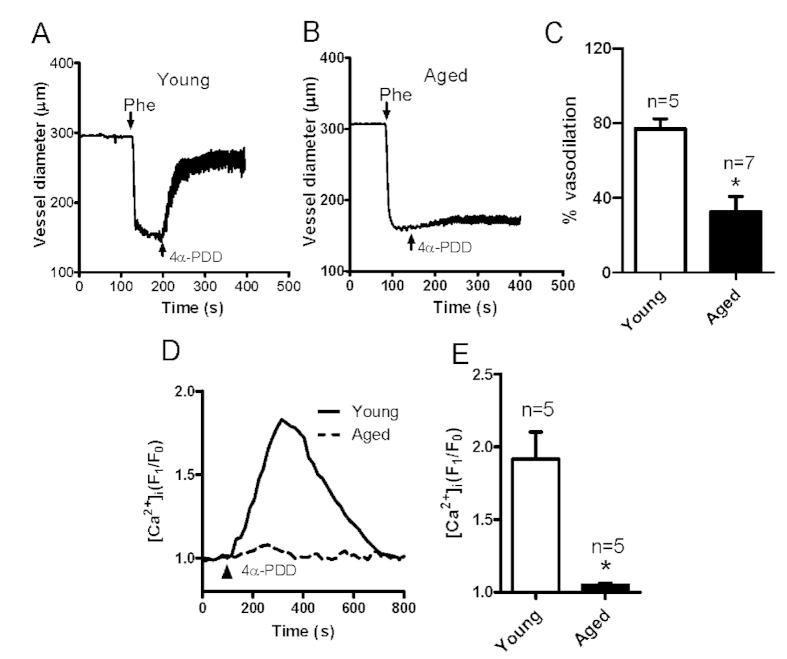
4α-PDD-stimulated mesenteric artery dilation and intracellular Ca^2+^ concentration ([Ca^2+^]_i_) rise in mesenteric artery endothelial cells (MAECs) derived from young and aged rats. (**A,B**) Representative traces showing the dilation response to a TRPV4 agonist (4α-PDD, 5 μM) in mesenteric arteries from young (**A**) and aged (**B**) rats. Vessels were preconstricted with phenylephrine (Phe) to approximately 60% of the preconstriction diameter. (**C**) Summarized data of the peak dilation stimulated by 4α-PDD in vessels from young and aged rats. (**D**) Representative traces of the 5 μM 4α-PDD-induced [Ca^2+^]_i_ rise in cultured primary MAECs from young and aged rats. E. Summarized data showing the 4α-PDD-stimulated [Ca^2+^]_i_ rise in primary cultured MAECs. Values are means ± SE (n = 5–7). ^*^P < 0.05, young (3 months old) vs. aged (22 months old) rats.

Many studies have shown that TRPV4 contributes to flow-induced vessel dilation and flow-induced Ca^2+^ rise^2^,^5^,^6^,^7^,^8^,^9^. To confirm the contribution of TRPV4 in flow-induced vessel dilation and [Ca^2+^]_i_ rise in our hands, we used the TRPV4 antagonist RN1734. We found that RN1734 pretreatment strongly reduced the flow-induced dilation of mesenteric arteries (Fig. 2A–C) as well as the flow-induced [Ca^2+^]_i_ rise in primary cultured MAECs derived from young rats (Fig. 2D,E), consistent with previous reports^6^,^7^,^19^,^20^. Next, to examine the involvement of TRPV4 in endothelium dysfunction, we investigated vessel dilation in response to application of the TRPV4 agonist 4α-PDD. Our data showed that applying 5 μM 4α-PDD elicited vessel dilation in Phe-precontracted mesenteric arteries derived from young rats (Fig. 3A,C). However, the 4α-PDD-induced vessel dilation was markedly decreased in mesenteric arteries obtained from aged rats (Fig. 3B,C). In addition, the 4α-PDD-induced [Ca^2+^]_i_ rise was nearly absent in aged primary cultured MAECs (Fig. 3D,E). These results indicated that the TRPV4-mediated flow response is impaired in aged vessels.

### TRPV4 expression level in MAECs decreases with aging

**Figure 4:**
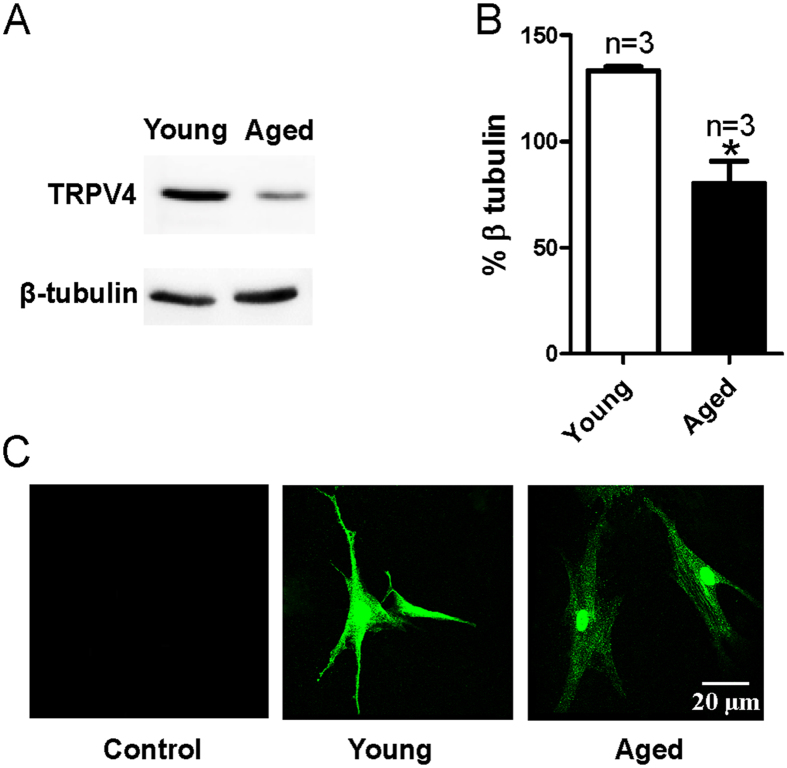
TRPV4 expression profiles in mesenteric artery endothelial cells (MAECs) derived from young and aged rats. Representative images (**A**) and summarized data (**B**) showing TRPV4 expression levels in cultured primary MAECs obtained from young and aged rats and evaluated using Western blotting assays. β-tubulin was used as a loading control. Protein levels are expressed as relative optical densities. Mean ± SE (n = 3). ^*^P < 0.05, young (3 months old) vs. aged (22 months old) rats. (**C)** MAECs are immunostained with an anti-TRPV4 antibody. In contrast to the control (omission of primary antibody), immunostaining for TRPV4 is detected in MAECs from young and aged rats.

Previous studies by others and our current study demonstrated a TRPV4-mediated endothelial Ca^2+^ influx and vasodilation in response to shear stress^6^,^7^,^19^,^20^. Additionally, our present data indicated that the TRPV4-mediated endothelial Ca^2+^ influx and shear stress-induced vasodilation were largely reduced with aging. To further explore the underlying mechanism, we evaluated TRPV4 expression levels in young and aged cultured primary MAECs. Our results showed that TRPV4 expression levels in primary cultured MAECs derived from aged rats were decreased compared with those from young rats by Western blotting (Fig. 4A,B). Our results from immunostaining assays further confirmed this finding (Fig. 4C).

### NO production in MAECs is reduced with aging

**Figure 5:**
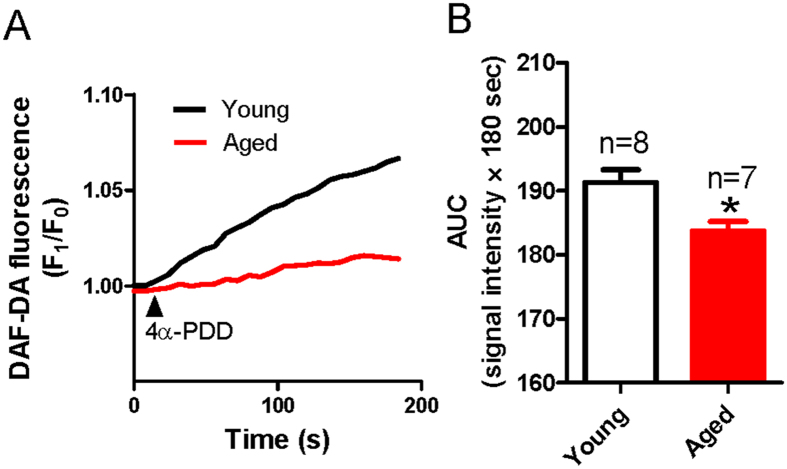
4α-PDD-stimulated nitric oxide (NO) production in mesenteric artery endothelial cells (MAECs) from young and aged rats. (**A**) Representative traces showing NO production in young and aged rat MAECs stimulated by 4α-PDD (5 μM) treatment. (**B**) Summarized data of the area under the curve (AUC) measured from the addition of 4α-PDD to 180 s. Values are means ± SE (n = 7–8). ^*^P < 0.05, young (3 months old) vs. aged (22 month old) rats.

The [Ca^2+^]_i_ rise in ECs may induce the production of NO, which acts on smooth muscle to induce vessel dilation^21^. To explore whether processes downstream of the TRPV4-induced [Ca^2+^]_i_ rise were altered with aging, we measured the amount of NO production stimulated by 4α-PDD in primary cultured MAECs. The NO production was evaluated by examining the 4-amino-5-methylamino-2′, 7′-difluorofluorescein (DAF-DA) fluorescence signal. We found that the 4α-PDD-stimulated NO production in MAECs derived from aged rats was significantly reduced compared with that in young rats (Fig. 5), indicating that impaired TRPV4 activity lowered NO production with aging.

### Increasing TRPV4 expression partially restores the deficits in flow-induced and TRPV4-mediated dilation in mesenteric arteries of aged rats

**Figure 6:**
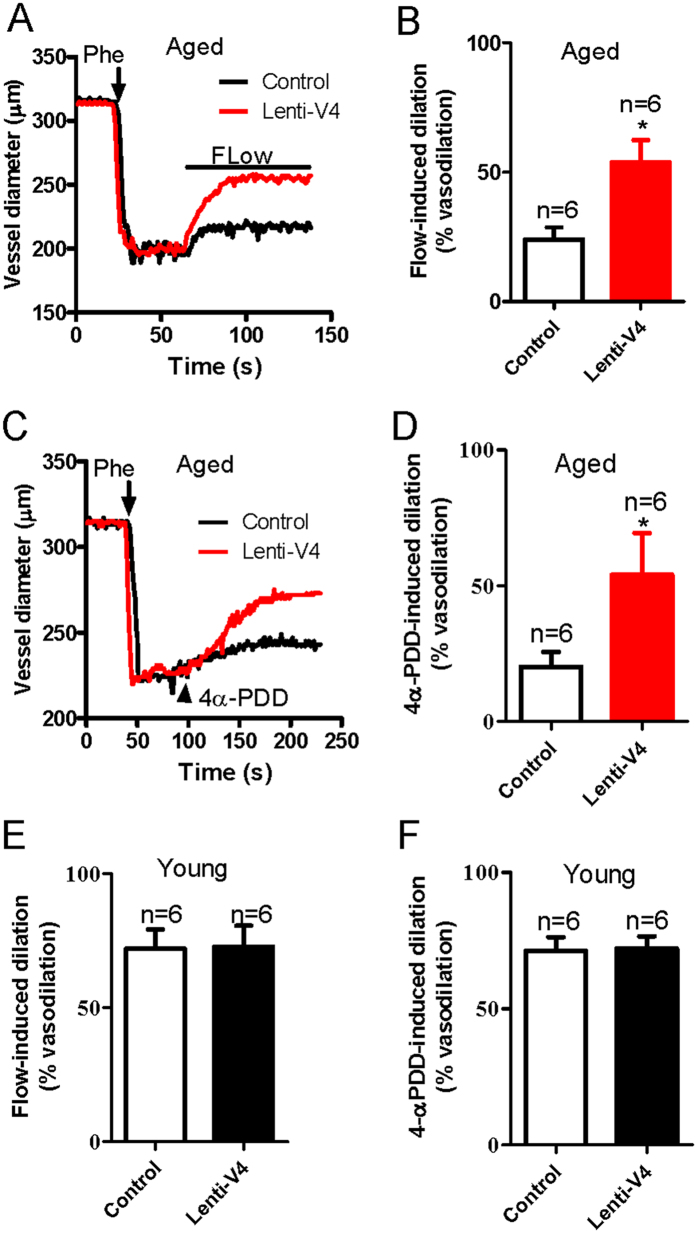
Flow- and 4α-PDD-induced mesenteric artery dilation in lenti-GFP or lenti-TRPV4 viral vector-treated young and aged rats. (**A**,**C**) Representative traces showing dilation responses to flow or 4α-PDD in mesenteric arteries derived from aged rats infected with lenti-GFP (Control) or lenti-TRPV4 (Lenti-V4) viral vectors. The vessel was preconstricted with phenylephrine (Phe) to approximately 60% of the preconstricted diameter. The solid bar above the trace shows the period of flow. (**B**,**D**) Summarized data showing the peak flow or 4α-PDD-induced dilation in lenti-GFP (Control) or lenti-TRPV4 (Lenti-V4) viral vector-treated vessels from aged rats. (**E**,**F**) Summarized data showing the peak flow or 4α-PDD-induced dilation in lenti-GFP (Control) or lenti-TRPV4 (Lenti-V4) viral vector-treated vessels from young rats. Values are mean ± SE (n = 6). ^*^P < 0.05, lenti-GFP (Control) vs. lenti-TRPV4 (Lenti-V4) viral vector-treated vessels.

**Figure 7:**
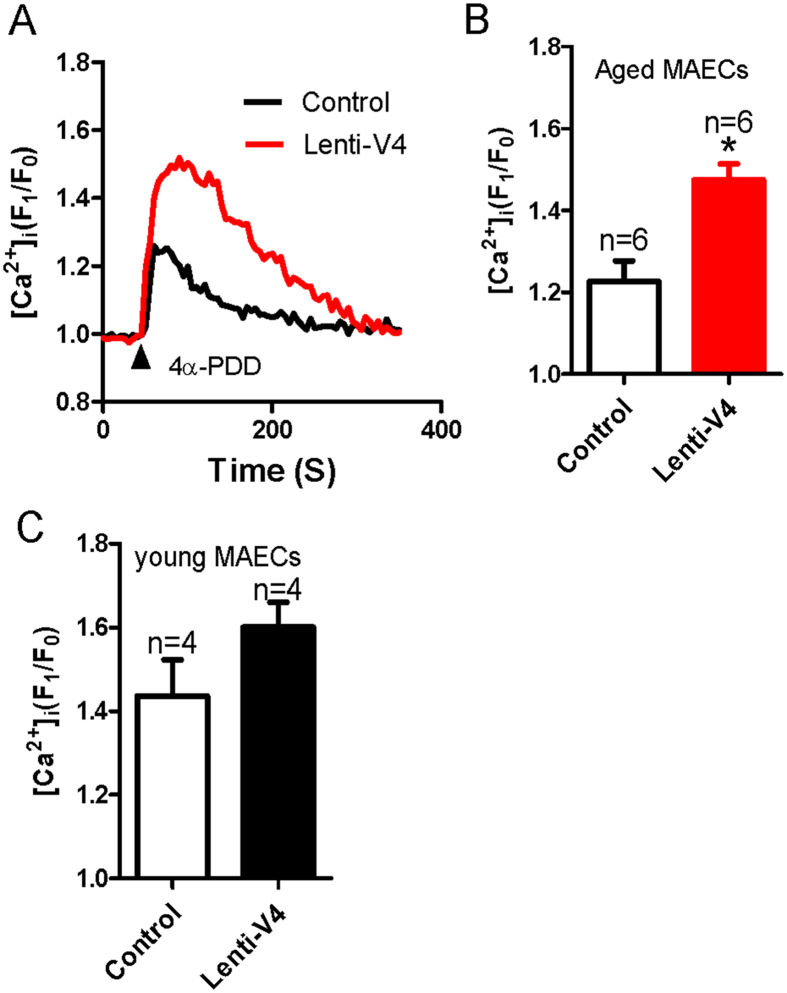
4α-PDD-induced intracellular Ca^2+^ concentration ([Ca^2+^]_i_) rise in lenti-GFP or lenti-TRPV4 viral vector-treated young and aged mesenteric artery endothelial cells (MAECs). (**A**) Representative traces showing 5 μM 4α-PDD-induced [Ca^2+^]_i_ rise in lenti-GFP (Control) or lenti-TRPV4 (Lenti-V4) viral vector-treated aged MAECs. (**B,C**) Summarized data showing the peak value of 4α-PDD-induced [Ca^2+^]_i_ rise in aged (**B**) and young (**C**) MAECs. Values are means ± SE (n = 4–6). ^*^P < 0.05, lenti-GFP (Control) vs. lenti-TRPV4 (Lenti-V4) viral vector-treated MAECs.

**Figure 8:**
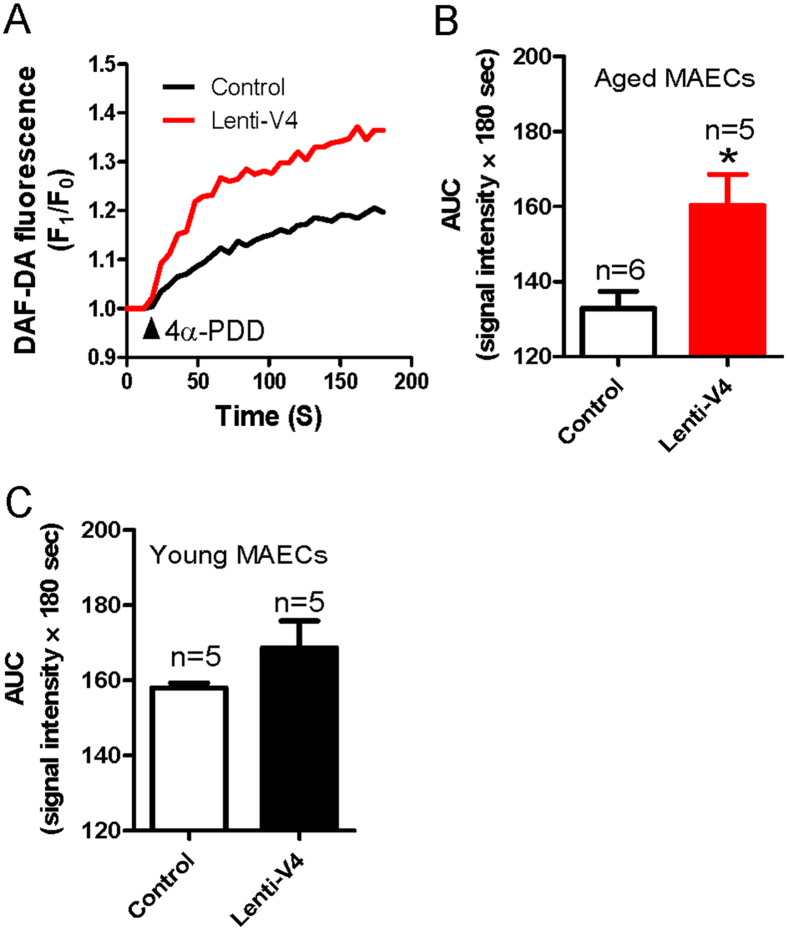
4α-PDD-induced nitric oxide (NO) production in lenti-GFP or lenti-TRPV4 viral vector-treated young and aged mesenteric artery endothelial cells (MAECs). (**A**) Representative traces showing NO production in lenti-GFP (Control) or lenti-TRPV4 (Lenti-V4) viral vector-treated aged MAECs stimulated by 4α-PDD (5 μM). (**B,C**) Summarized data of the area under the curve (AUC) measured from the addition of 4α-PDD to 180 s in aged (**B**) and young (**C**) MAECs. Values are means ± SE (n = 5–6). ^*^P < 0.05, lenti-GFP (Control) vs. lenti-TRPV4 (Lenti-V4) viral vector-treated MAECs.

The results above show that the reduction in the flow-induced and TRPV4-mediated dilation of mesenteric arteries from aged rats was associated with a decrease in the TRPV4 expression level in these vessels. Therefore, we next elevated TRPV4 expression in ECs by generating a lentiviral construct carrying *TRPV4* (lenti-TRPV4) and administering it intravenously to young and aged rats. Successful lentiviral transduction was confirmed by the observation of lenti-GFP fluorescence in the endothelial layer of the arteries. We found that the GFP fluorescence was enhanced significantly in the endothelial layer of lenti-GFP transfected mesenteric arteries (Supplementary Fig. 2A). To further confirm the transduction efficiency of lenti- TRPV4, we tested expression level of TRPV4 in young and aged MAECs. The results showed that TRPV4 expression level was statistically increased in aged MAECs, but only slightly increased in young MAECs when both young and aged MAECs were treated by lenti-GFP and lenti-TRPV4 (Supplementary Fig. 2B,C). More interestingly, we found that the impaired flow-evoked and 4α-PDD-induced dilation in mesenteric arteries from lenti-TRPV4-treated aged rats were significantly rescued compared with those from lenti-GFP-treated aged rats (Fig. 6A–D). By contrast, lenti-TRPV4 transfection did not significantly affect flow- or 4α-PDD-induced dilation in mesenteric arteries derived from young rats (Fig. 6E,F). In addition, lenti-TRPV4 transfection did not affect the vasoconstriction induced by application of a high K^+^ solution in young and aged rats (Supplementary Fig. 3). [Ca^2+^]_i_ rise and NO production, as the upstream signal in MAECs, importantly contribute to the vessel dilation. To uncover the underlying mechanism of lenti-TRPV4-increased flow-evoked dilation, we examined the effect of 4α-PDD on [Ca^2+^]_i_ rise and NO production in lenti-TRPV4 transduced young and aged MAECs. Consistent to the result of flow-induced dilation, [Ca^2+^]_i_ rise and NO production were significantly enhanced or recovered by lenti-TRPV4 transduction in aged MAECs, but only slightly increased in young MAECs without significant difference (Figs 7 and 8). These results suggest that decreased TRPV4 expression may be a primary cause of endothelial function deficits in aged rats.

## Discussion

In the present study, we investigated TRPV4 expression level alterations in MAECs and the functional role of TRPV4-mediated flow-induced mesenteric artery dilation in aged rats. Our five major findings include the following. (1) The results from pressure myography studies showed that vessel dilation induced by flow or the TRPV4 activator 4α-PDD was significantly decreased in mesenteric arteries of aged rats compared with that in young rats. (2) The [Ca^2+^]_i_ rise induced by flow or 4α-PDD was decreased in primary cultured MAECs derived from aged rats. (3) The immunoblotting and immunostaining data showed a significant decrease in TRPV4 expression levels in MAECs from aged relative to young rats. (4) Aging lowered the NO production induced by 4α-PDD in MAECs. (5) Compared with that in lenti-GFP-treated aged rats, the impaired flow- or 4α-PDD-induced vessel dilation was significantly rescued in mesenteric arteries isolated from lenti-TRPV4-treated aged rats. Taken together, these data demonstrated an age-related impairment in the TRPV4-mediated [Ca^2+^]_i_ rise in MAECs and in the flow-induced mesenteric artery dilation.

The mesenteric arteries are proximal resistance vessels that substantially contribute to the majority of blood flow resistance in the circulation. Shear stress induced by blood flow acts on ECs to modulate smooth muscle tone and consequently blood vessel lumen diameter^22^,^23^. ECs function in mechanotransduction, acting as an interface between flowing blood and the vessel wall. In ECs, sensing a flow-induced stimulus is the earliest stage in shear stress-induced mechanotransduction. The present study showed that flow-induced vessel dilation in mesenteric arteries derived from aged rats was significantly decreased compared with that in young rats. Therefore, vascular endothelial dysfunction occurs during aging and may be a critical risk factor for the development of cardiovascular diseases, such as atherosclerosis and hypertension.

The present study showed that the flow-stimulated [Ca^2+^]_i_ rise was decreased in aged MAECs. The [Ca^2+^]_i_ rise in vascular ECs serves as an important initial event in flow-induced vessel dilation and leads to the production and release of dilation factors, such as NO, prostacyclin, and EDHFs. NO contributes to blood pressure regulation, and the NO synthase inhibitor L-NAME increases blood pressure^24^. Therefore, if the agonist- or shear stress-induced [Ca^2+^]_i_ rise is blunted, the endothelium-dependent vessel dilation may be impaired. Previous studies have demonstrated that NO and the prostacyclin mediation of endothelium-dependent dilation were impaired in several types of vascular beds with aging^25^. However, the underlying molecular targets remained unknown. Our findings suggested that the reduced flow-stimulated [Ca^2+^]_i_ rise contributed to the decreased flow-induced vessel dilation in the mesenteric arteries of aged rats. More interestingly, we identified TRPV4 as a crucial molecular target responsible for endothelium dysfunction in aging.

Multiple TRP channels participate in flow-induced Ca^2+^ influx, including TRPV4^2^,^6^,^19^,^20^, TRPP2^26^, TRPV4-TRPC1^27^, TRPV4-TRPP2^8^,^28^ and TRPV4-TRPC1-TRPP2^9^. However, in overexpressing systems, previous studies have shown that flow only evokes the [Ca^2+^]_i_ rise in TRPV4-expressing HEK293 cells, but not in HEK293 cells expressing TRPC1 or TRPP2^7^,^8^. Our results are consistent with those from these previous studies, further indicating that TRPV4 is essential in shear stress-induced endothelial Ca^2+^ influx and vasodilation. To reveal the pathological relevance of TRPV4 in the decreased flow response of aged mesenteric arteries, we examined the 4α-PDD-induced [Ca^2+^]_i_ rise and vessel dilation. Our data demonstrated that the 4α-PDD-induced response was significantly decreased in aged mesenteric arteries. The NO production induced by the TRPV4-mediated [Ca^2+^]_i_ rise was also decreased in aged MAECs. The immunoblotting and immunostaining data also showed that the TRPV4 expression level in aged MAECs was significantly reduced compared with that in young rats. These results suggested that an attenuated TRPV4-mediated [Ca^2+^]_i_ rise may result from the decreased TRPV4 expression level in aged MAECs. This hypothesis was confirmed using lenti-TRPV4 constructs to restore TRPV4 expression in aged rats. Impaired flow- or 4α-PDD-induced dilation was significantly recovered by restoring TRPV4 expression levels in aged rats. In addition to TRPV4, other TRP family members, such as TRPC3, have been suggested to play a role in flow-induced vasodilation in rat small mesenteric arteries^29^. Along with TRP channels, other molecular elements involved in flow stimulus sensing have been identified in ECs, including the cytoskeleton, tyrosine kinase receptors, caveolae, G proteins, and cell-matrix and cell-cell junction molecules^23^,^30^,^31^. Thus, multiple factors may contribute to the age-related impairment of ECs, and our data indicate that TRPV4-mediated Ca^2+^ influx is one of the contributors to age-related impairment in MAECs.

In conclusion, our data suggested that the impairment of TRPV4-mediated Ca^2+^ signaling in ECs contributed to the age-related decrease of flow-induced vessel dilation in mesenteric arteries. The present study sheds new light on the potential value of TRPV4 for clinical treatment of age-related vascular system diseases. But here we only provide a clue of the pathological relevance of TRPV4. Future study on age-related vascular system diseases, for example hypertension or atherosclerosis animal models, may benefit this important issue.

## Methods

### Cell preparation and culture

All animal experiments were conducted in accordance with the U.S. National Institutes of Health (NIH publication No. 8523) and were approved by the Animal Experimentation Ethics Committee of Anhui Medical University.

Primary cultured MAECs were isolated from male Sprague Dawley rats (weight: 250–300 g; aged: 3 or 22 months) and maintained as previously described. Briefly, the rats were killed by CO_2_ asphyxiation, and the heart was perfused with phosphate-buffered saline (PBS; 140 mM NaCl, 3 mM KCl, and 25 mM Tris; pH 7.4) to remove circulating blood. The small intestine was dissected. All vein branches of the mesenteric bed were rapidly excised, and the remaining arterial branches were digested with 0.02% collagenase type I (Sigma) in PBS for 45 min at 37 °C. The digested samples were centrifuged at 1600 × *g* for 5 min, and the pelleted cells resuspended in DMEM supplemented with 10% FBS, 100 μg/mL penicillin, and 100 U/mL streptomycin. One hour after plating the cells, the medium was changed to remove non-adherent cells. The remaining adherent MAECs were cultured at 37 °C with 5% CO_2_ for 3–5 days. The identity of the primary cultured rat MAECs was verified by immunostaining using an antibody against von Willebrand factor. The results showed that 98% of cells were of endothelial origin (Supplementary Fig. 1).

### [Ca^2+^]_i_ measurement

[Ca^2+^]_i_ measurements were performed as previously described^4^,^9^. Briefly, MAECs seeded on rectangular coverslips were loaded with 10 μM Fluo-8/AM and 0.02% pluronic F-127 for 30 min. The coverslip with the cells was mounted in a parallel plate flow chamber. The flow was initiated by pumping normal physiological saline solution (NPSS) containing 140 mM NaCl, 5 mM KCl, 1 mM CaCl_2_, 1 mM MgCl_2,_ 10 mM glucose, 5 mM HEPES, and 1% BSA (pH 7.4). The fluorescence signals were recorded and analyzed using a Leica TCS SP5 confocal laser system, and [Ca^2+^]_i_ changes were displayed as a ratio of fluorescence (F_1_/F_0_). The endothelial cells were challenged with 4α-PDD (5 μM) or the shear stress induced by flow that was approximately 5 dyne/cm^2^.

### Vessel diameter measurement using a pressure myography system

Lenti-TRPV4 or lenti-GFP vector virus (1.0 × 10^7^ copies) were intravenously injected in young and aged rats. Third-order mesenteric arteries were dissected from the rats 72 hrs later. Successful lentiviral transduction was confirmed by the observation of lenti-GFP fluorescence in the endothelial layer of the arteries. Vessel diameter measurements were performed as previously described^9^. Briefly, the mesenteric arteries were cut into segments 2–3 mm in length and then mounted in a pressure myograph chamber (Danish MyoTechnology, Denmark, model 110P) with two glass micropipettes. Both cannulation pipettes were connected to independent reservoirs set to the same level to ensure equal pressures and no flow in the vessel segments. The pressure myograph chamber was heated to 37 °C and filled with 5 mL of Krebs solution, which was continuously bubbled with a gas mixture of 95% O_2_ and 5% CO_2_ to maintain a pH of 7.4. After a 60 min equilibration period at 50 mmHg intraluminal pressure, the contractile function of the vessel was tested using a 60 mM K^+^ solution. Following washout and a 30-min equilibration period, the artery was precontracted to approximately 60% of its initial vessel diameter by using 1–3 μM phenylephrine (Phe) to achieve a similar constriction among the various artery diameters. The flow was initiated by perfusing Krebs solution containing 1% BSA into the vessel lumen, or by adding 4α-PDD to the chamber to elicit vessel dilation. The external diameter of the artery was recorded continuously with a CCD camera using MyoView software (version 1.1 P, 2000, Photonics Engineering). Before each experiment, the endothelium viability was assessed by dilation with 1 μM acetylcholine after the preconstriction with 1 μM Phe. Intact vessels failing to achieve at least 80% of the maximal relaxation average were assumed to be damaged and were discarded.

### Immunoblotting

Proteins were extracted from primary cultured MAECs lysates with detergent extraction buffer containing 1% Nonidet P-40, 150 mM NaCl, 20 mM Tris-HCl (pH 8.0) and protease inhibitor cocktail tablets (Sigma). Following protein transfer to a PVDF membrane, the membrane was incubated with a primary anti-TRPV4 antibody (1:200, Santa Cruz) at 4 °C overnight. The primary antibody was visualized with a horseradish peroxidase-conjugated secondary antibody (1:1000, GE Healthcare) that was processed with an enhanced chemiluminescence detection system (GE Healthcare). The optical density of each blot was normalized to that of β-tubulin (1:200, Santa Cruz), which was analyzed within the same lane, and presented as relative optical density.

### Immunostaining

Immunostaining was performed as previously described^7^,^9^ with MAECs that were seeded onto circular coverslips and fixed with 4% formaldehyde for 10 min, followed by permeabilization with 0.1% Triton X-100 in PBS. The cells were blocked with 2% BSA at room temperature for 60 min and then incubated with primary anti-TRPV4 antibody (1:100) at 4 °C overnight. Following incubation, samples were washed three times with PBS and incubated with goat anti-rabbit IgG secondary antibody conjugated to Alexa Fluor 488 (1:200) for 60 min at room temperature. The samples were then washed three times with PBS, the coverslips were mounted using 90% glycerol in PBS, and the fluorescence was detected with a Leica TCS SP5 confocal laser system.

### Measurement of NO production in primary cultured MAECs

MAECs seeded on circular coverslips were loaded with 2 μM 4-amino-5-methylamino-2′, 7′-difluorofluorescein (DAF-FM DA; Molecular Probes) at room temperature for 10 min. The fluorescence intensity excited at 495 nm and emitted at 515 nm was determined using a Leica TCS SP5 confocal laser system. The MAECs were stimulated with 5 μM 4α-PDD in NPSS. Changes in intracellular NO levels were displayed as a ratio of fluorescence (F_1_/F_0_).

### Statistical analysis

Student’s *t*-tests were used for comparisons of means from two groups, with *P* values less than 0.05 deemed significant.

## Additional Information

**How to cite this article**: Du, J. *et al*. Increasing TRPV4 expression restores flow-induced dilation impaired in mesenteric arteries with aging. *Sci. Rep.* **6**, 22780; doi: 10.1038/srep22780 (2016).

## Supplementary Material

Supplementary Information
