# Supplementary material for: Increasing TRPV4 expression restores flow-induced dilation impaired in mesenteric arteries with aging
Source: Sci Rep. 2016 Mar 7;6:22780. doi: 10.1038/srep22780 (PMC4780030; doi:10.1038/srep22780)
Supplement: Supplementary Information [file srep22780-s1.pdf]

Increasing TRPV4 expression restores flow-induced dilation impaired in mesenteric arteries with aging

Juan Du[1,#,*], Xia Wang[1,#], Jie Li[1,#], Jizheng Guo[1], Limei Liu[3], Dejun Yan[1], Yunyun Yang[1], Zhongwen Li[1], Jinhang Zhu[1], Bing Shen[1,2,*]

[1]Department of Physiology, School of Basic Medicine, Anhui Medical University, Hefei, Anhui 230032, China.

[2]Central laboratory of molecular and cellular biology of School of Basic Medicine, Anhui Medical University, Hefei, Anhui 230032, China.

[3]Department of Physiology and Pathophysiology, Peking University Health Science Center, Peking 100191, China.

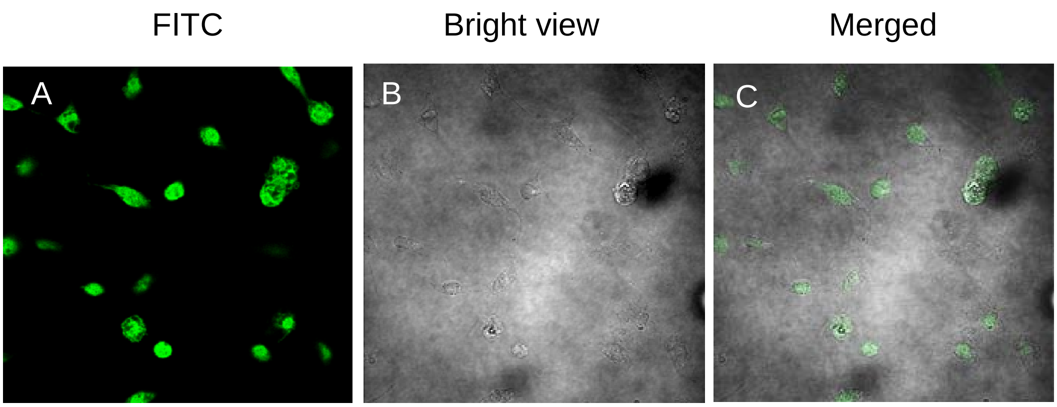

Supplementary Figure 1. Representative images showing immunostaining of cultured primary mesenteric artery endothelial cells using an antibody against von Willebrand factor.

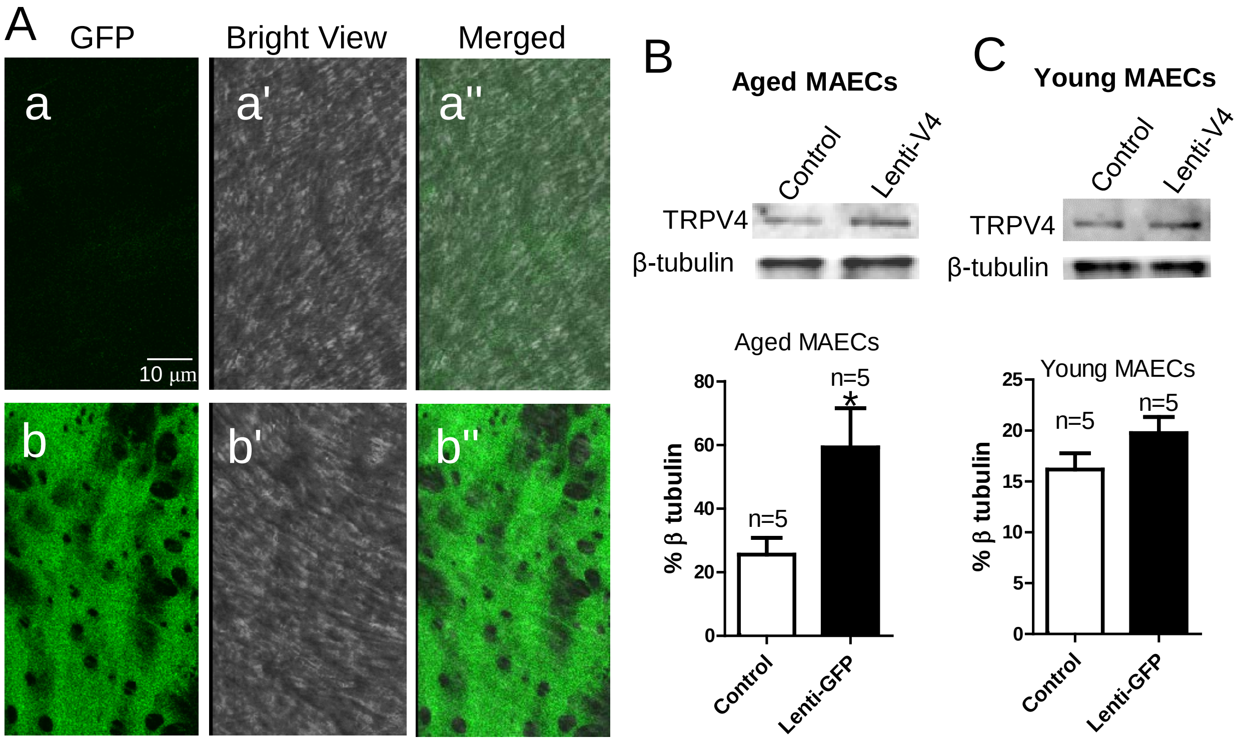

Supplementary Figure 2. Effect of lenti-GFP and lenti-TRPV4 transfection in the expression of green fluorescent protein (GFP) in mesenteric artery endothelium and TRPV4 protein in primary cultured mesenteric artery endothelial cells (MAECs) from young and aged rats. A. Representative images showing the signal of green fluorescence indicating GFP in the endothelial layer of mesenteric artery from young rat. **a**-**a''**. Lenti-empty vector infection. **b**-**b''**. Lenti-GFP infection. **B** and **C.** Shown are representative images (upper) and data summary (lower) of immunoblotting experiments for TRPV4 in lenti-TRPV4 transfected young and aged MAECs. Proteins were extracted from primary cultured MAECs lysates after 72 h of virus infection. Immunoblots with anti-β-Tubulin antibody showed that equal amounts of protein were loaded onto each lane. Values are mean ± SE (n = 5). *P < 0.05, lenti-GFP (Control) vs. lenti-TRPV4 (Lenti-V4) viral vector-treated MAECs.

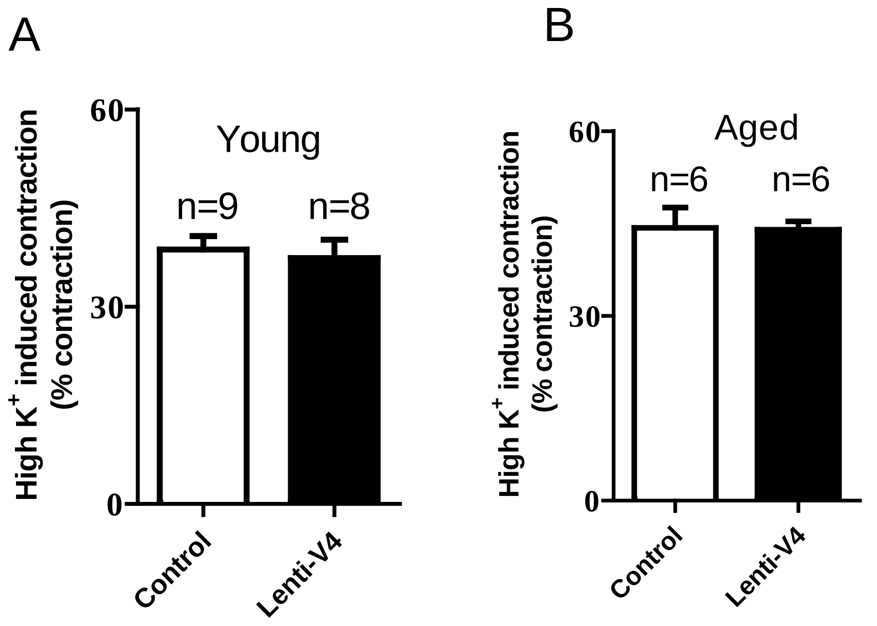

Supplementary Figure 3. High K^+ solution-induced mesenteric artery contraction. A and **B**. Summarized data showing the peak value of high K^+ solution-induced contraction. Values are mean ± SE (n = 6-9). *P < 0.05, lenti-GFP (Control) vs. lenti-TRPV4 (Lenti-V4) viral vector-treated vessels.
